# Manipulation of immunodominant dengue virus E protein epitopes reduces potential antibody-dependent enhancement

**DOI:** 10.1186/1743-422X-9-115

**Published:** 2012-06-18

**Authors:** Holly R Hughes, Wayne D Crill, Gwong-Jen J Chang

**Affiliations:** 1 Arboviral Diseases Branch, Division of Vector-Borne Diseases, Centers for Disease Control and Prevention, U.S. Department of Health and Human Services, 3150 Rampart Road, Fort Collins, CO, 80521, USA

**Keywords:** Dengue virus, Vaccine, Antibody-dependent enhancement, Dengue hemorrhagic fever, Cross-reactive antibody, Immune refocusing

## Abstract

**Background:**

Dengue viruses (DENV) are the most important arboviruses of humans and cause significant disease. Infection with DENV elicits antibody responses to the envelope glycoprotein, predominantly against immunodominant, cross-reactive, weakly-neutralizing epitopes. These weakly-neutralizing antibodies are implicated in enhancing infection via Fcγ receptor bearing cells and can lead to increased viral loads that are associated with severe disease. Here we describe results from the development and testing of cross-reactivity reduced DENV-2 DNA vaccine candidates that contain substitutions in immunodominant B cell epitopes of the fusion peptide and domain III of the envelope protein.

**Results:**

Cross-reactivity reduced and wild-type vaccine candidates were similarly immunogenic in outbred mice and elicited high levels of neutralizing antibody, however mice immunized with cross-reactivity reduced vaccines produced significantly reduced levels of immunodominant cross-reactive antibodies. Sera from mice immunized with wild-type, fusion peptide-, or domain III- substitution containing vaccines enhanced heterologous DENV infection *in vitro*, unlike sera from mice immunized with a vaccine containing a combination of both fusion peptide and domain III substitutions. Passive transfer of immune sera from mice immunized with fusion peptide and domain III substitutions also reduced the development of severe DENV disease in AG129 mice when compared to mice receiving wild type immune sera.

**Conclusions:**

Reducing cross-reactivity in the envelope glycoprotein of DENV may be an approach to improve the quality of the anti-DENV immune response.

## Background

Dengue virus (DENV) is a mosquito-borne flavivirus of global public health concern as two-fifths of the world’s population live in DENV endemic/epidemic regions [1]. Infection with any one of the four closely related serotypes (DENV-1 to −4) can result in a wide range of clinical symptoms from subclinical; to the classic febrile illness, dengue fever (DF); to dengue hemorrhagic fever (DHF) characterized by a plasma leakage syndrome and its most severe form, life threatening dengue shock syndrome (DSS). Infection with one DENV serotype confers life-long homotypic immunity, while heterotypic immunity is transient [2]. Epidemiological evidence indicates severe DHF/DSS is correlated with a second DENV infection [3,4]. Although the mechanism leading to severe disease is not fully understood, it is thought to be multifactorial. Two non-mutually exclusive immunopathological mechanisms to describe the events leading to DHF/DSS are antibody-dependent enhancement of infection (ADE) [5] and the involvement of cross-reactive memory T cells [6,7] through the process of original antigenic sin [7]. ADE occurs when cross-reactive weakly- or non-neutralizing antibodies bind to viruses and form antibody-virus complexes that enhance infection of Fcγ receptor bearing cells resulting in an increase in virus load. Higher viremia has been shown to be associated with severe disease [8].

DENV vaccine development has been a WHO priority for over forty years. Through considerable effort, several vaccine candidates are undergoing human clinical trials [9] with possible licensure being accomplished in the next five years. With the global expansion of DENV and recent autochthonous transmission of DENV in Key West, Florida [10], the need for a safe and efficacious vaccine is in increased demand.

The envelope (E) glycoprotein is responsible for eliciting the majority of the protective DENV antibody response. The E protein covers the virion surface arranged in anti-parallel dimers containing three structural and antigenic domains [11]. Studies in mice and recently in humans demonstrate that E protein domain I (EDI), the central domain contains virus specific and cross-reactive, predominantly non-neutralizing epitopes; EDII, the dimerization domain contains the internal fusion peptide (EDII_FP_) and overlapping immunodominant, group cross-reactive, weakly-neutralizing epitopes [12,13]; EDIII has an immunoglobulin-like fold, that is involved with receptor binding, and contains serocomplex cross-reactive and serotype-specific potently neutralizing epitopes [14-16].

Recent studies of sera from DENV infected patients suggest group cross-reactive antibodies recognizing residues in the EDII_FP_ constitute a major proportion of the anti-E antibody response [17,18] while a small proportion of the anti-E antibody response appears to be responsible for protection [19,20]. The dominant production of high avidity, weakly or non-neutralizing antibodies could compete with neutralizing antibodies recognizing overlapping epitopes through steric interference [21] and promote severe disease through ADE. Structural studies of immature West Nile virus virions suggest cross-reactive weakly-neutralizing antibodies recognizing the EDII_FP_ bind with high affinity to immature and partially mature virions but do not bind effectively to mature virions [22]. Both murine and human monoclonal antibodies directed against the EDII_FP_ have been shown to enhance DENV infection leading to lethal disease in the AG129 mouse model [23-25]. These data suggest broadly cross-reactive antibodies could promote ADE of DENV infection by increasing infectivity of low infectious, partially mature or immature virions [26]. These studies lead us to speculate that dengue infection elicits a predominantly poor quality immune response strongly skewed toward non-protective potentially pathogenic antibodies.

In this study, we describe the initial preclinical evaluation of a next generation DENV vaccine [27]. We developed cross-reactivity reduced DENV-2 DNA vaccine candidates, based upon a previously described DENV-2 DNA vaccine expressing prM and E proteins that self-assemble to form virus-like particles (VLP) and was shown to passively protect mice from DENV-2 challenge [28]. These candidates are engineered with specific substitutions in immunodominant E protein B cell epitopes [19]. By introducing specific substitutions into the EDII_FP_ (at G106 and L107) and serocomplex cross-reactive eptiopes of EDIII (at K310, E311 and P364), we constructed a series of cross-reactivity reduced DENV-2 DNA vaccine candidates attempting to dampen or eliminate the induction of cross-reactive, enhancing antibodies recognizing weakly and non-neutralizing epitopes thereby potentially improving the quality of the antibody response elicited by immunization. Wild-type (WT), unmodified DENV-2 prM/E plasmid vaccine is compared with cross-reactivity reduced DENV-2 prM/E plasmids containing substitutions knocking out antibody recognition of epitopes in the EDII_FP_, due to the immunodominant nature of this epitope; in the serocomplex cross-reactive epitopes of EDIII, since the severity of disease correlated with secondary infection is a dengue phenomenon; and by combining both of these regions in an attempt to maximize cross-reactivity reductions based on the observation of antibody classes recognizing inter-domain epitopes [19,29]. The cross-reactivity reduced vaccines stimulate high levels of serotype-specific neutralizing antibody, similar to that of the unmodified WT vaccine. Importantly, unlike sera from WT vaccinated mice, sera from one cross-reactivity reduced vaccine-immunized mouse group did not enhance heterologous DENV infection *in vitro*, and significantly reduced homologous ADE *in vivo*. Thus, the B cell epitope modifications introduced into the E glycoprotein can reduce the potential for vaccine-induced ADE and severe DENV disease.

## Results

### Vaccine construction and characterization

Cross-reactivity reduced plasmids containing substitutions in the EDII_FP_ (at G106 and L107) and in EDIII (at K310, E311 and P364) were selected by screening plasmid secreted virus-like particles (VLPs) with a well-characterized panel of murine monoclonal antibodies (MAbs) and looking for significant reductions or ablation of VLP recognition by cross-reactive MAbs representing four distinct reactivity classes: group, subgroup, complex and subcomplex cross-reactive antibodies, while retaining reactivity by serotype-specific protective neutralizing antibodies [19]. The cross-reactivity reduced vaccines selected for further study were: pVD2iG106R/L107D (RD) containing substitutions in EDII_FP_; pVD2iK310E/E311R/P364R (ERR) containing substitutions in EDIII; and pVD2iG106R/L107D/K310E/E311R/P364R (RDERR) a combination of both EDII_FP_ and EDIII substitutions (Table 1). The RD vaccine candidate significantly reduced or ablated the reactivity of the most broadly, flavivirus cross-reactive MAbs such as 4G2. The ERR candidate reduced or knocked out the reactivity of predominately DENV sub-complex cross-reactive MAbs, but also sub-group reactive MAbs not affected by the fusion peptide substitutions of the RD vaccine. The two examples of these were MAbs 20 and 5–1 raised against DENV-2 and Japanese encephalitis virus (JEV), respectively which each recognizes both DENV-2 and JEV. The RDERR vaccine construct reduced the reactivity of the majority of the cross-reactive MAbs, including all of those reduced by either the RD or the ERR constructs alone, plus additional MAbs not significantly reduced by either the EDII_FP_ or EDIII substitutions such as the DENV complex cross-reactive non-neutralizing MAb D3-5 C9-1. Importantly, potently neutralizing DENV-2 serotype-specific MAb 3H5 reactivity was not altered by any of the introduced substitutions. 3H5 is the prototype serotype-specific neutralizing MAb and recognizes an epitope shared by many DENV-2 specific, protective MAbs [30].

**Table 1:** **MAb reactivities for DENV-2 VLP mutants**^**1**^

**MAb:**	**MHIAF**	**4G2**	**6B6C-1**	**4A1B-9**	**23-1**	**23-2**	**20**	**5-1**	**5-2**	**1B7-5**	**D3-5 C9-1**	**1A1D-2**	**9D12**	**10A4D-2**	**1B4C-2**	**3 H5**
**CR**^**2**^**:**	**Poly-clonal**	**group**	**group**	**group**	**group**	**group**	**Sub grp.**	**Sub grp.**	**Sub grp.**	**comp.**	**comp.**	**Sub comp.**	**Sub comp.**	**Sub comp.**	**Sub comp.**	**Type-spec.**
**Virus:**	D2	D2	SLEV	MVEV	WNV	JEV	JEV	D2	JEV	D3	D4	D2	D1	D2	D2	D2
**VLP construct**																
WT DENV-2^3^	≥6.0	≥6.0	≥6.0	5.1	≥6.0	≥6.0	≥6.0	≥6.0	≥6.0	≥6.0	5.1	≥6.0	≥6.0	≥6.0	>4.2	≥6
RD^5^	100	**<0.1**	**<0.1**	**<3**	**0.2**	**0.2**	100	**3**	100	100	25	100	100	**6**	nd^4^	50
ERR	100	100	100	150	100	100	**<0.1**	**<0.1**	100	100	100	**0.2**	**0.1**	100	nd	100
RDERR	100	**<0.1**	**0.2**	**<0.8**	**<0.1**	**0.2**	**<0.1**	**<0.1**	100	100	**1.5**	**0.1**	**0.4**	100	**<1.3**	100

^1^Percent of wild-type (WT) DENV-2 VLP reactivity for MAbs exhibiting varying levels of cross-reactivity (CR) and selected from different flaviviruses.

^2^ MHIAF is polyclonal murine ascitic fluid; group CR antibodies recognize all viruses of the four major pathogenic flavivirus serocomplexes; sub-group CR MAbs recognize all or some members of two or more different flavivirus serocomplexes (e.g., MAbs 20, 5–1 and 5–2 recognize DENV-2 and JEV, JEV and DENV-2 and JEV, DENV-1 and −2 respectively); comp. and sub-comp. CR MAbs recognize all four DENV complex viruses or a subset thereof respectively, and type-specific MAbs recognize only DENV-2.

^3^ MAb reactivities for wild-type (WT) DENV-2 VLP are presented as inverse log_10_ antigen-capture ELISA endpoint values and all mutant VLPs as percent of remaining reactivity compared to WT.

^4^ nd denotes not determined.

^5^Mutant VLPs containing amino acid substitutions in the EDII_FP_ G106R/L107D (RD), in EDIII K310E/E311R/P364R (ERR), or in both the EDII_FP_ and EDIII G106R/L107D/K310E/E311R/P364R (RDERR).

Reproduced in part from: Crill WD, Hughes HR, Delorey MJ, Chang GJ (2009) Humoral immune responses of dengue fever patients using epitope-specific serotype-2 virus-like particle antigens. PLoS ONE 4: e4991.

### Cross-reactivity reduced vaccines elicit DENV-specific neutralizing antibody

**Figure 1:**
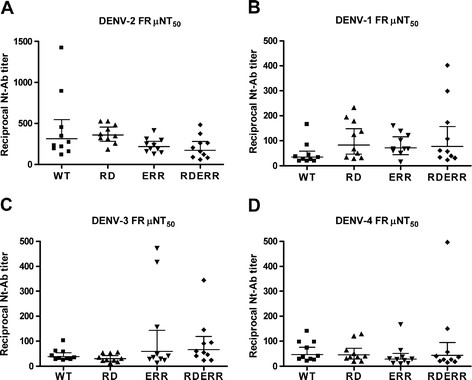
**Cross-reactivity reduced vaccines elicit DENV specific neutralizing IgG.** (**A**) DENV-2 16681 neutralizing-antibody (Nt-Ab) titers elicited by WT and cross-reactivity reduced vaccines as determined by 50% FRμNT. (**B**) DENV-1 56BC94/95 Nt-Ab titers. (**C**) DENV-3 116RC1396 Nt-Ab titers. (**D**) DENV-4 H241 Nt-Ab titers. Data are expressed as GMT +/− 95%CI. for n = 10. Kruskal-Wallis test and Dunn’s post test was used to analyze data. p < 0.05 were considered significant; single asterisk, p < 0.05.

Outbred Swiss-Webster mice (n = 10) were immunized at 0 and 5-weeks. Although vaccine responses are expected to be more variable in outbred than in inbred mice, we specifically chose outbred mice because they are a better model for correlating human vaccine responses than inbred mice. Ten weeks post vaccination we evaluated the immunogenicity of WT and cross-reactivity reduced vaccines using a focus-reduction micro-neutralization assay (FRμNT) against DENV-2 (Figure 1A). All vaccinated mice produced DENV-2 specific neutralizing antibody with mean reciprocal 50% endpoint geometric mean titers ranging from 173 to 361. None of the mean neutralizing antibody titers from the cross-reactivity reduced immunized mice differed significantly from that of WT immunized mice. There was however, a significant effect of vaccine treatment on DENV-2 neutralizing antibody titer as determined by a non-parametric Kruskal-Wallis test (p = 0.03), and a Dunn’s post test revealed RD immunized mouse sera, that had the highest mean neutralizing antibody titer, was significantly different from RDERR vaccinated sera that had the lowest titer (p = 0.02). In mice immunized with cross-reactivity reduced vaccines containing substitutions in EDIII, ERR and RDERR, there was a trend toward a decrease in homologous DENV-2 neutralization. The apparent reduction in neutralization induced by EDIII substitution containing vaccines could be due to reductions in EDIII cross-reactive neutralizing antibody, some of which can neutralize potently, e.g., MAb 1A1D-2 or 9D12 [16,19,31]. There were no significant differences in mean 50% FRμNT titers as determined by a non-parametric Kruskal-Wallis test between WT and any of the cross-reactivity reduced vaccine immunized mouse sera when tested against DENV-1 (p = 0.08), DENV-3 (p = 0.18), and DENV-4 (p = 0.39) (Figure 1B-D).

### Characterization of the epitope-specific immune profile

We utilized an epitope-specific IgG ELISA [19] to determine the quantity and quality of the cross-reactivity reduced vaccine DENV-2 antibody response (Table 2). Both murine and human monoclonal antibodies directed against the EDII_FP_ have been shown to enhance DENV infection leading to lethal disease in the AG129 mouse model [23-25]. Similar to studies examining DENV infected human sera [17,19], the vaccines containing an unmodified EDII_FP_, WT and ERR vaccine, elicited a large proportion of the total E response directed against EDII_FP_ epitopes (average 66.8% and 81.5%, respectively), which was highly variable between individual outbred mouse sera. This data agrees with the monoclonal antibody mapping of Table 1 where WT and ERR VLPs maintain full reactivity toward group cross-reactive antibodies. Cross-reactivity reduced plasmids containing EDII_FP_ substitutions (RD and RDERR) elicited almost no antibodies recognizing the WT EDII_FP_ epitope (Table 1; means of 0.3% and <1.0%, and p = 0.005 and p = 0.002 compared to WT for RD and RDERR respectively). Despite this altered antibody profile, total DENV-2 IgG endpoint mean titers elicited by WT and all cross-reactivity reduced plasmids were similar and ranged from 4.6 × 10^4^ to 1.6 × 10^5^ (n = 10 each, p = 0.22).

**Table 2:** WT and cross-reactivity reduced vaccines epitope-specific IgG antibody response

**Vaccine**	**Specific Epitope**^**1**^	**Endpoint mean**^**2**^	**Endpoint range**	**% response mean**^**3**^	**% response range**^**3**^
WT	E	9.8 × 10^4^	<1.0 × 10^2^ -2.9 × 10^5^	100.0	100.0
	EDII_FP_	5.9 × 10^4^	<1.0 × 10^2^-1.2 × 10^5^	66.8	45.3-93.6
RD	E	1.6 × 10^5^	2.9 × 10^2^-9.7 × 10^5^	100.0	100.0
	EDII_FP_	4.3 × 10^5^	7.1 × 10^4^-1.2 × 10^6^	0.3^4^	<1-3.2
ERR	E	1.2 × 10^5^	<1.0 × 10^2^-5.2 × 10^5^	100.0	100.0
	EDII_FP_	9.1 × 10^4^	<1.0 × 10^2^-1.8 × 10^5^	81.2	58.9-100
RDERR	E	4.6 × 10^4^	<1.0 × 10^2^-3.1 × 10^5^	100.0	100.0
	EDII_FP_	7.1 × 10^5^	<1.0 × 10^2^-2.3 × 10^6^	<1^4^	<1

^1^ Epitopes targeted by specific antibody populations. E denotes antibodies recognizing all E-protein epitopes. EDII_FP_ denotes broadly cross-reactive WT epitopes of the E domain II fusion peptide.

^2^ Endpoint titers determined with WT and epitope knock-out antigens, thus representing immunoglobulins not recognizing epitopes targeted by the knock-out antigen [19] (see methods).

^3^ Percentages below limit of detection are expressed as <1.

^4^ The percent IgG recognizing these epitopes was significantly lower than for WT immunized mice as determined by a non-parametric Kruskal-Wallis test and Dunn’s multiple comparison tests.

### Cross-reactivity reduced vaccine candidates exhibit reduced ADE potential *in vitro*

**Figure 2:**
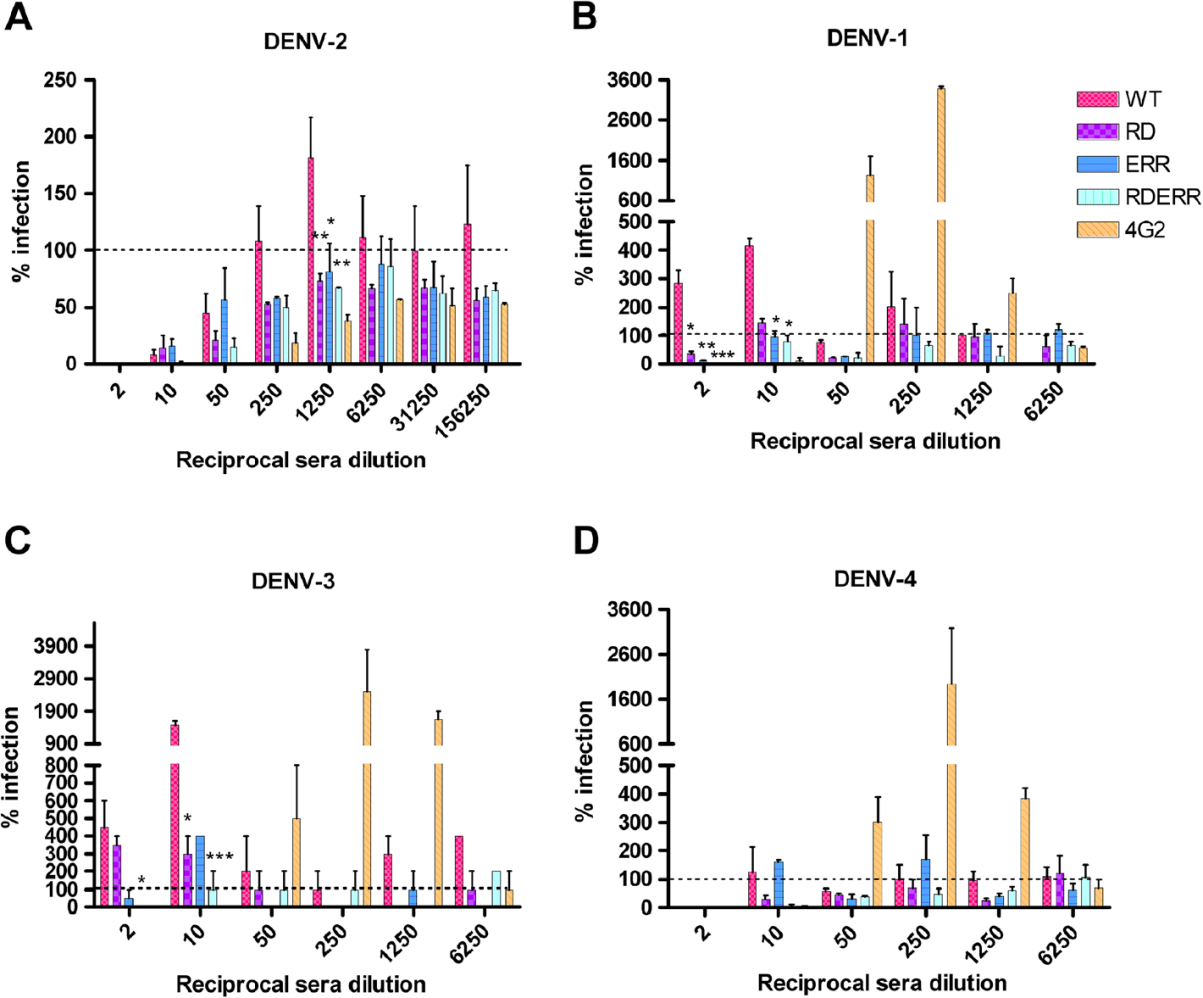
**Cross-reactivity reduced vaccines have reduced potential to participate in ADE*in vitro*compared to WT vaccine.** (**A**) DENV-2 16681 enhancement reported as percent infection compared to the virus input control (dashed line) (**B**) DENV-1 56BC94/95 percent infection. (**C**) DENV-3 116RC1396 percent infection. (**D**) DENV-4 H241 percent infection. All data represent the mean+/−s.e.m. of two independent experiments. Two-way ANOVA were performed on square-root transformed data with a Bonfferoni post test; p < 0.05 were considered significant; single asterisk, p < 0.05; two asterisks, p < 0.01.

We next compared the three different cross-reactivity reduced vaccine constructs to determine whether altered cross-reactive antibody profiles can reduce the potential for ADE *in vitro*. FcRII bearing human K562 cells were infected with DENV alone, with DENV-immune complexes formed in the presence of mouse sera from WT or cross-reactivity reduced vaccine immunized mice, or with DENV-immune complexes formed in the presence of EDII_FP_ recognizing MAb 4G2. The K562 cell line has previously been shown to support ADE of infection as a relevant model for DHF [32-37]. Sera from WT immunized mice significantly enhanced the infection of homologous DENV-2 at relatively high serum dilutions whereas cross-reactivity reduced vaccine immune mouse sera did not enhance DENV-2 ant any serum concentration tested. WT vaccinated sera enhanced at a peak titer of 1:1,250 compared to RD (p = 0.01), ERR (p = 0.021), and RDERR (p = 0.01) (Figure 2A).

In heterologous ADE assays, sera from WT vaccinated mice typically enhanced at the lowest serum dilutions in the assay, RD and ERR vaccinated sera enhanced DENV-1 and −3 at some serum dilutions, and only RDERR vaccinated sera lacked statistically significant enhancement of any DENV serotype at any dilution (Figure 2B-D). 4G2 control antibody enhanced DENV-1, -3, and −4 strongly between 1:50 and 1:1,250 dilutions but did not enhance DENV-2 at the dilutions we tested. This observation is consistent with both *in vitro* and *in vivo* studies demonstrating that this DENV-2 raised antibody enhanced infection *in vitro* at dilutions greater than 200,000, beyond the dilutions of this high titer ascites fluid tested here [38,39]. 4G2 has also been demonstrated to neutralize and even protect from DENV-2 challenge at higher concentrations [23,25,38,39]. WT vaccinated sera significantly enhanced DENV-1 infection at the lowest dilution tested (1:2) compared to RD (p = 0.05), ERR (p = 0.01), and RDERR (p = 0.001), and also enhanced DENV-1 infection at a 1:10 dilution compared to ERR (p = 0.04) and RDERR (p = 0.03) (Figure 2B). WT vaccinated sera significantly enhanced DENV-3 infection at a dilution of 1:2 compared to only RDERR (p = 0.029) and at a dilution of 1:10 compared to RD (p = 0.026) and RDERR (p = 0.006) (Figure 2C), whereas ERR vaccinated sera enhancement was not significantly different from that of WT vaccinated sera. This suggests an important role of the immunodominant EDII_FP_ targeting antibody response in the enhancement of severe disease because RD and RDERR vaccines do not produce antibodies which recognize WT EDII_FP_ while ERR immunized mice produced similar proportions of EDII_FP_ recognizing antibody as WT (Table 2). None of the serum from vaccinated mice significantly enhanced DENV-4 infection (Figure 2D). Thus, the *in vitro* enhancement analysis indicated that the combination of substitutions in EDII_FP_ and EDIII_CR_ incorporated into the RDERR plasmid elicited the highest quality antibody response as only RDERR immune sera lacked DENV enhancing capabilities.

**Figure 3:**
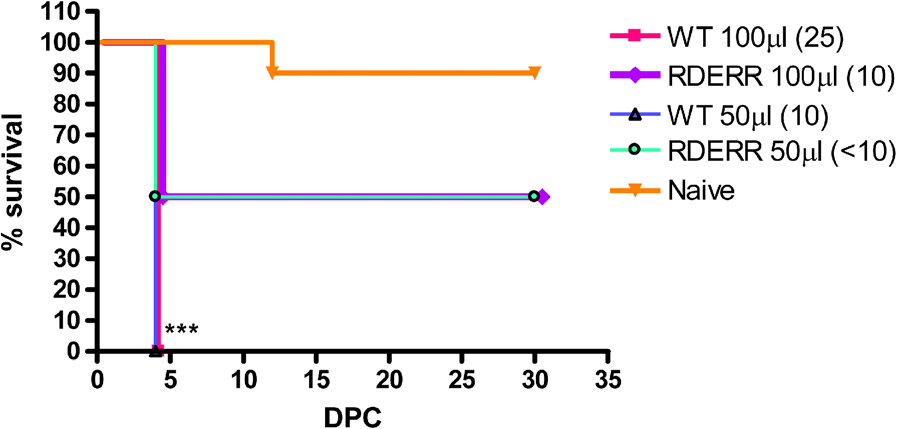
**Cross-reactivity reduced vaccine increases survival from ADE*in vivo.*** Survival of AG129 mice passively transferred 100 or 50 μL of pooled WT or RDERR immune sera from homologous ADE with 4.2 × 10^4^ ffu of DENV-2 S221. Kaplan-Meyer survival analysis (n = 10). p < 0.008 were considered significant due to Bonferonni adjusted alpha for multiple comparisons; three asterisk p < 0.0001.

### Cross-reactivity reduced vaccine candidate reduces potential ADE *in vivo*

Because of the high DENV-2 neutralizing antibody titer, the combination of reduced cross-reactive antibodies with the lack of any *in vitro* enhancement, we selected RDERR as the best cross-reactivity reduced vaccine candidate to examine potential improvements in the quality of anti-DENV antibody response by the *in vivo* ADE assay using the published AG129 mouse model [23,40].

Unfortunately, there are no published DENV-1, -3 or −4 mouse adapted dengue strains available to us that can cause vascular leak-associated enhanced disease in AG129 mice, making heterologous *in vivo* ADE difficult to examine. Previous studies have described the capability of mouse-adapted DENV-2 S221 strain to produce DHF-like disease via ADE in AG129 mice [23] allowing us to utilize this virus to test if reductions in cross-reactive antibody populations of passively transferred RDERR vaccinated Swiss Webster mouse sera can reduce homologous ADE *in vivo*. We passively transferred 100 μl or 50 μl of pooled sera from WT immunized, RDERR immunized, or naïve Swiss mice into AG129 mice (n = 10 each treatment). Sixteen hours post serum transfer, 2 randomly selected mice per group were tail bled and their circulating neutralizing antibody titers were determined. Eighteen hours post transfer, all mice were challenged with a sub-lethal dose (4.2 × 10^5^ ffu) of DENV-2 S221 (Figure 3). Only one of ten mice receiving passively transferred naïve sera died 13 days post challenge. One-hundred percent of mice receiving either 50 or 100 μl of WT immune sera (FRμNT_50_ = 1:10 and 1:25, respectively) succumbed to hemorrhagic disease 4–5 days following challenge. These animals exhibited the classic pathology associated with enhanced DENV-2 S221 disease including intestinal hemorrhage, vascular leak, and rapid onset mortality [23] (p < 0.0001, compared to naïve controls, Bonferroni α = 0.008). Only 50% of mice receiving either 50 or 100 μl of RDERR immune sera (FRμNT_50_ <1:10 and 1:10, respectively) succumbed to lethal disease (p = 0.05, compared to naïve controls, Bonferroni α = 0.008). Although only 50% of mice from both RDERR immunized serum transfer groups survived challenge, the survival of RDERR serum transfer mice was significantly higher than WT (p = 0.0005, Bonferroni α = 0.008), implying that modification of EDII_FP_ and EDIII_CR_ epitopes can reduce the induction of potentially pathogenic cross-reactive IgG and thereby improve the quality of the immune response to limit ADE *in vivo.*

## Discussion

DENV infection elicits primarily a poor quality immune response directing a high proportion of antibody against non-protective, potentially pathogenic epitopes and only a small proportion against potently neutralizing and protective epitopes. In this report we have shown the manipulation of these potentially pathogenic epitopes as a vaccine strategy [41] that can reduce ADE *in vitro* and *in vivo.* Immunization of mice demonstrated that knocking out immunodominant cross-reactive epitopes in the EDII_FP_ and EDIII did not significantly effect DENV-2 neutralization, however the removal of these epitopes dramatically altered the vaccine induced antibody repertoire and sera from vaccinated mice shows reduced ADE *in vitro* and reduced lethal enhancement of DENV *in vivo.* Such a strategy could be applicable to other DENV vaccine formats, however, it might not be applicable to DENV live-attenuated vaccines because mutations in the EDII_FP_ can be lethal [42].

Our findings demonstrate that by introducing targeted amino acid substitutions into immunodominant cross-reactive E protein epitopes of a DENV-2 DNA vaccine that we can significantly reduce the induction of antibodies associated with immune enhancement, that are stimulated from these epitopes [17]. Epitope-specific IgG ELISA revealed that cross-reactivity reduced vaccinated mouse sera contained dramatically reduced proportions of cross-reactive EDII_FP_ specific antibodies, had reduced potential for ADE *in vitro*, and when passively transferred into AG129 mice exhibited a significant reduction in hemorrhagic vascular leak associated mortality compared to passively transferred WT vaccinated mouse sera. WT vaccinated mouse sera however, contained EDII_FP_ specific antibodies in proportions similar that of naturally infected human sera. The capability of EDII_FP_ recognizing antibodies to induce severe DENV disease similar to DHF via ADE has been demonstrated in the AG129 mouse model [23-25]. Cross-reactivity reduced vaccines with alterations in EDII_FP_ (RD and RDERR) elicited almost no antibodies which recognize the WT EDII_FP_ epitope (Table 2), and therefore are less likely to participate in ADE *in vivo*. Supporting this conclusion, The RD and RDERR vaccine candidates exhibited the least ADE potential *in vitro* with RDERR vaccinated mouse sera being the only cross-reactivity reduced candidate that completely lacked *in vitro* ADE across all dilutions and serotypes of DENV.

One objective of this study was to dampen the immunodominance of the EDII_FP_ through alteration of EDII_FP_ amino acids. Although studies with immunotoxin and other therapeutic proteins have shown amino acid substitutions can dampen the immunogenicity of B cell epitopes [43], RD and RDERR vaccines did elicit antibodies which recognize the mutated EDII_FP_ epitope, in similar proportions as those recognizing the WT EDII_FP_ from WT vaccinated mice (67% and 73% respectively). These results suggest that although the specificity of this antigenic region was completely altered, the immunodominance was not reduced by the RD substitutions. These data also suggest that instead of dampening the recognition of the modified RD EDII_FP_ epitope by B cell receptors, the substitution of G106R might allow for anchoring of the B cell receptor, as suggested by studies identifying arginine as a main anchor residue in protein-protein and antibody-antigen interactions [44,45] Though RD-induced novel antibodies do not recognize WT DENV (Table 2) and therefore would not participate in ADE in a natural infection, the production of novel antibodies is potentially concerning and ongoing studies include mapping of amino acid residues responsible for immunodominance and subsequent alterations in an effort to ablate the antigenicity of this epitope.

Severe DHF is associated with secondary DENV infections in older children and adults, or primary infections in infants. To evaluate and compare the different cross-reactivity reduced vaccine constructs for altered antibody profiles, we measured Fc receptor-dependent ADE of WT and cross-reactivity reduced vaccinated mouse serum *in vitro*. Sera from mice vaccinated with WT DENV-2 DNA vaccine maximally enhanced DENV-2 infection of K562 cells at a dilution four times higher than the average DENV-2 neutralization titer. These results show that as the antibody concentration decreases the proportion of neutralizing antibodies becomes insufficient to neutralize virus, allowing for ADE [46]. This phenomena is similar to primary DHF in infants where maternal antibody titers correlated to infant age at onset of severe disease [32] when maternally derived anti-DENV IgG maintained reactivity with DENV virions but could not neutralize virus [47]. None of the cross-reactivity reduced vaccine constructs enhanced homologous DENV-2 replication at any dilution tested.

WT vaccinated mouse serum significantly enhanced both DENV-1 and DENV-3 replication in human K562 cells at 1:2 serum dilution (the lowest dilutions tested due to the availability of testing serum), which is the closest simulation to undiluted sera and the most relevant for ADE from natural exposure. Sera from RD and ERR vaccinated mice were also able to significantly enhance DENV-1 and DENV-3 at some serum dilutions. Conversely, sera from RDERR vaccinated mice did not enhance any DENV serotype at any dilution tested. This finding suggests the possible involvement of the adjacent antigenic regions of EDII_FP_ and EDIII in the development of enhancing antibodies as concurrent modifications to both regions were necessary to eliminate ADE. Our MAb mapping of cross-reactivity reduced vaccine constructs [19] points to a possible antibody class to explain this phenomenon. MAb D3-5 C9-1 is a DENV-4 derived, complex cross-reactive, non-neutralizing antibody. D3-5 C9-1 exhibited only minor reductions in reactivity for the RD plasmid derived VLPs, no reductions for ERR VLPs, but greater than 98% reduction in reactivity compared to WT for the RDERR plasmid derived VLPs. This implies that D3-5 C9-1 recognizes an epitope overlapping EDII_FP_ and EDIII of adjacent monomers within the E dimer. The DENV complex cross-reactivity and lack of any neutralizing capability of this MAb suggest that antibodies recognizing epitopes similar to D3-5 C9-1 could be an important DENV disease enhancing antibody class.

In an *in vivo* DENV disease AG129 mouse model, we demonstrated that DENV-2 RDERR vaccination reduced mortality from homologous ADE compared to WT vaccine. While 100% of mice receiving WT immune sera succumbed to lethally enhanced disease, only 50% of mice receiving RDERR immune sera developed terminal disease. The apparent decrease in ADE by RDERR immune sera raises the question whether reduced ADE is due to shifting [25] or blunting of the enhancement curve? Due to the limited availability of sera, we only tested homologous enhancement at two passively transferred serum volumes. Mice receiving RDERR immune sera of either volume had higher survival compared to mice receiving WT immune sera. Mice receiving 100 μl RDERR immune sera had post transfer FRμNT_50_ titer = 1:10, the same post-transfer titer as mice receiving 50 μl of WT vaccinated serum (Figure 3). Thus, RDERR post serum transfer neutralizing antibody titer fell within that of the maximal enhancing range of WT immune sera and yet maintained significantly higher survival, suggesting RDERR immune sera blunts the enhancement curve. In addition, mice immunized with either WT or RDERR plasmids elicited similar levels of total IgG recognizing DENV-2 E (Table 2), suggesting the distinct differences in the quality of the immune response and reduced potentially pathogenic antibody in RDERR was responsible for differences in ADE.

In a homologous enhancement scenario, we would not expect cross-reactivity reduced immunized mice to exhibit completely reduced ADE because many unaltered antibody classes from RDERR immune sera can still react with the homologous E protein epitopes of the challenge virus. Similar phenomena have been demonstrated with passively transferred, protective and potently neutralizing antibodies [24,46]. Moreover, the role of flavivirus non-neutralizing type and strain-specific antibodies [48] in ADE is not well characterized.

Similarly, the residual enhancement observed with passively transferred RDERR vaccinated mouse sera could be due to prM/M targeting antibodies [37,39]. Recent studies have begun to expand upon the prescient historical deductions of Henchal et al., regarding the potential importance of prM antibodies in the human polyclonal immune response to DENV infection and their ability to enhance the infection of immature virus particles potentially exacerbating secondary DENV disease [18,26,37,39,49]. DNA vaccines direct the expression of prM/M and E proteins which self-assemble and are secreted as immunogenic VLP. A potential limitation of this study is that we did not characterize the particulate nature of the cross-reactivity reduced DENV-2 vaccine antigens and although they do form a pelletable antigen *in vitro*, this might not be identical to the VLPs which have been characterized with our WT plasmids [28]. Regardless the physical nature of the antigens secreted by mutant plasmids is identical or not, the critical outcome of immune response is the most important parameter being measured. Previous studies by our group have shown the expression of prM [50,51] by our DNA plasmids and vaccination with VLPs elicits anti-prM as wells as anti-E antibodies [50]. Although the current report did not evaluate the presence of prM targeting antibodies in the antibody response toward our cross-reactivity reduced vaccines or their ability to enhance DENV infection, the manipulation of the EDII_FP_ and EDIII epitopes were effective in significantly reducing the ADE capability of serum from vaccinated mice *in vitro* and *in vivo*. As the role of prM antibody associated ADE becomes clearer, the same approach used here to ablate immunodominant enhancing E protein epitopes can be applied to prM. Studies to identify and characterize prM epitopes and to examine their inclusion into our DENV cross-reactivity reduced vaccine constructs are currently ongoing.

## Conclusions

Vaccinology has returned many successful endeavors including vaccines against smallpox, yellow fever, and childhood diseases such as pertussis and measles. Classical methods of vaccine development have been vital in the progression of this field and public health. However, these classical approaches have not been successful for some important and highly variable or multi-strain pathogens such as DENV, enteroviruses, HIV, and hepatitis C virus. For vaccination to become a reality for such diseases or improving current vaccines like influenza and foot and mouth disease virus, new vaccine approaches need to be developed taking into account the unique epitope hierarchy in each pathogen and tailoring immunogens to elicit an appropriate protective immune response [52-55]. Increasing the induction of broadly cross-reactive yet protective immune responses for HIV, influenza, and hepatitis C virus, or reducing pathogenic cross-reactive responses for example, in DENV may become possible with these and other similar approaches. In this report, by manipulating potentially pathogenic, immunodominant epitopes, we have altered the antibody response to reduce the potential for ADE and begin to scratch the surface of immune refocusing approaches in DENV vaccine development.

## Materials and methods

### Vaccines

pVD2i plasmid directing the expression of DENV-2 16681 prM and ectodomain E proteins was derived from pCBD2-2 J-2-9-1 which been previously characterized and described in detail [13,19,28]. Characterization of DENV-2 DNA plasmids with substitutions in the EDII_FP_ and EDIII have been described and characterized previously [19]. Vaccines were manufactured by Aldevron.

### Mice

Swiss Webster mice (DVBD) (n = 10) were vaccinated 100 μg intramuscularly at 0 and five weeks. Sera collected 10 weeks post vaccination. Swiss Webster mice (n = 20) were vaccinated 100 μg of WT or RDERR vaccine at 0 and 8 weeks and sera collected at 12 weeks and pooled for passive transfer. Six week old AG129 mice (DVBD) (n = 10) were passively transferred Swiss immune sera (i.p.). 18 hours post transfer mice were challenged with 4.2 × 10^5^ ffu of DENV-2 S221[23] (in kind from S. Shresta) (i.p.) to determine homologous enhancement. Animal experiments were approved by IACUC.

### Virus neutralization

FRμNT technique was utilized as previously described [19], briefly: Vaccinated mouse sera (n = 10) were diluted 1:10, heat inactivated, titrated 2-fold to the volume of 40 μL, and 320 virus pfu/40 μL was added to each dilution. Plates were then incubated for 1 hr at 37°C, 5% CO_2_. After incubation, 25 μl serum and virus suspensions were transferred back to Vero cell monolayer containing plates. These Vero cell plates were incubated at 37°C, 5% CO_2_ for 45 minutes rocking every 5 minutes to allow for virus infection. Barry’s Ye Lah overlay media containing 6% sodium bicarbonate and 1% Carboxymethylcellulose sodium salt (Fluka biochemical) was added and plates were incubated at 37°C, 5% CO_2._ Incubation times were as follows: DENV-2 (16681) and DENV-4 (H241): 48 hr; DENV-1 (56BC94/95), DENV-3 (116RC1396): 70 hr. FRμNT titers were calculated for each virus relative to a back titration. Exact FRμNT titers were modeled using Graph Pad Prism version 4 sigmoidal dose response (variable slope) formula. Values are the average of two independent replicates.

### Epitope-specific IgG ELISA

The same IgG-capture ELISA protocol described previously [19] was used with a few modifications. We utilized previously characterized epitope-specific knock-out antigens [19]. Sera were assayed for the presence of E-specific immunoglobulins with IgG antigen capture ELISAs (GAC-ELISA). All VLP antigen concentrations were standardized using anti-DENV-2 polyclonal rabbit sera capture and MHIAF detection ELISA and methods described previously [19]. Antigen concentrations were standardized at an OD of 1.5, within the region of antigen excess near the upper asymptote of the sigmoidal OD curve. GAC-ELISA was performed as previously described with some modifications [56]. Briefly, Immulon II HB flat-bottom 96-well plates were coated overnight at 4°C with goat anti-human IgM or IgG (Kirkegaard & Perry Laboratories) and blocked with StartBlock. Vaccinated mouse sera (n = 10) were diluted 1:100 and serially titrated in wash buffer, added to wells and incubated at 37°C for 90 min. WT DENV-2, cross-reactivity reduced DENV-2 and negative control antigens were diluted appropriately in wash buffer tested against each serum sample in duplicate and incubated overnight at 4°C. DENV-2 virus-infected MHIAF was diluted in 5% milk/PBS and incubated for 1 h at 37°C. Horseradish-peroxidase conjugated goat anti-mouse IgG was diluted in 5% milk/PBS, 50 μl, added to wells and incubated for 1 h at 37°C. Bound conjugate was detected with TMB substrate and plates incubated at room temperature for 8 min. The reaction was stopped with 2 N H_2_SO_4_ and OD was measured at *A*_450_. OD values were modeled as non-linear functions of the log10 sera dilutions using a Gaussian non-linear regression in Graph Pad Prism version 4.0 and endpoint dilutions determined as the titer where the OD value equaled two-times the OD value of the test serum reacted against normal antigen.

EDII_FP_ epitope-specific IgG percentages were calculated as previously described [19] with minor modifications. Briefly, the EDII_FP_ epitope specific IgG percentage for vaccinated mice were calculated by dividing the IgG endpoint titer obtained with specific knock-out antigens by the endpoint titer obtained by WT antigen on the same sera, subtracting this value from 1.0 and multiplying by 100. EDII_FP_ epitope-specific percentages were calculated as 100 x [1.0-(RD antigen endpoint/WT antigen endpoint)]. The percentage of IgG targeting the modified RD epitope were calculated as 100 x [1.0-(WT antigen endpoint/RD antigen endpoint)]

### *In vitro* antibody-dependent enhancement

Heat inactivated mouse sera were pooled, diluted, and titrated. DENV (DENV-1 56BC94/95, DENV-2 16681, DENV-3 116RC1396, DENV-4 H241) was added to each dilution and incubated for 1 hour at 37 °C. K562 cells (MOI = 0.5) were added to the antibody-virus complexes and incubated 2 hours. After infection, cells were centrifuged, supernatants removed, resuspended in RPMI media (10% FBS) and plated. DENV infection alone was used as virus control; MAb 4G2 was used as a positive control.

### Focus titration assay

Supernatants from DENV infected K562 cells were collected 48 hours post infection, clarified by centrifugation, and serially titrated. 2.47 × 10^4^ Vero cells in DMEM (10% FBS) were added to 96-well black plates (Corning/Costar), and infected with each titration in duplicate. Cells were incubated, overlaid, acetone fixed, immunostained and counted as described for FRμNT. ffu/mL of each sera dilution was calculated as percent of DENV only control.

### Statistical analysis

Neutralization data are scatter plots of individuals, with geometric mean titer and 95% confidence interval. Data are analyzed with a non-parametric Kruskal-Wallis test and Dunn’s post test. ADE data are displayed as means+/−s.e.mand were square-root transformed transformed to achieve homogenous variances (Leven’s test) and normality (Kolmogrovo-Smirnov test). Transformed neutralization data was analyzed with two-way ANOVA and Bonferroni post-test (p < 0.05 were considered significant). Survival curves were analyzed with a Kaplan-Meyer analysis. Where appropriate, p < 0.008 were considered significant due to Bonferroni adjusted α for multiple comparisons. Statistical analysis performed with SAS 9.2.

## Competing interests

The author(s) declare that they have no competing interests.

## Authors’ contributions

HRH designed, performed and analyzed experiments, and wrote the paper. WDC designed and analyzed experiments, and critically reviewed the paper. GJJC designed and analyzed experiments, provided materials and reagents, and critically reviewed the paper. All authors read and approved the final manuscript.
